# Gas-Phase Ambient Air Contaminants Exhibit Significant Dioxin-like and Estrogen-like Activity *in Vitro*

**DOI:** 10.1289/ehp.8496

**Published:** 2005-12-29

**Authors:** Gail P. Klein, Erin M. Hodge, Miriam L. Diamond, Amelia Yip, Tom Dann, Gary Stern, Michael S. Denison, Patricia A. Harper

**Affiliations:** 1 Department of Pharmacology and; 2 Department of Geography, Centre for Urban Health Initiatives, University of Toronto, Toronto, Ontario, Canada; 3 Analysis and Air Quality Division, Environment Canada, Ottawa, Ontario, Canada; 4 Freshwater Institute, University of Manitoba, Winnipeg, Manitoba, Canada; 5 Department of Environmental Toxicology, University of California, Davis, California, USA; 6 Program in Developmental Biology, Hospital for Sick Children, Toronto, Ontario, Canada

**Keywords:** AHR, air pollution, CALUX, complex mixtures, endocrine disruption, ER, PAH

## Abstract

Several adverse health effects, such as respiratory and cardiovascular morbidity, have been linked to exposure to particulate matter in ambient air; however, the biologic activity of gas-phase ambient organic air contaminants has not been examined as thoroughly. Using aryl hydrocarbon receptor (AHR)–based and estrogen receptor (ER)–based cell bioassay systems, we assessed the dioxin-like and estrogenic activities of gas-phase organic ambient air contaminants compared with those of particulate-phase contaminants using samples collected between seasons over 2 years from an urban and a rural location in the Greater Toronto Area, Canada. The concentration of the sum (∑) of polycyclic aromatic hydrocarbons, which was highest in the gas phase, was 10–100 times more abundant than that of ∑polychlorinated biphenyls, ∑nitro-polycyclic aromatic hydrocarbons, and ∑organochlorine pesticides, and 10^3^ to 10^4^ times more abundant than ∑polychlorinated dibenzo-*p*-dioxins/dibenzofurans. Gas-phase samples induced significant AHR- and ER-dependent gene expression. The activity of the gas-phase samples was greater than that of the particulate-phase samples in the estrogen assay and, in one case, in the AHR assay. We found no strong associations between either summer or winter seasons or urban or rural locations in the relative efficacy of the extracts in either the ER or AHR assay despite differences in chemical composition, concentrations, and abundance. Our results suggest that mechanistic studies of the health effects of ambient air must consider gas and particulate phases because chemicals present in both phases can affect AHR and ER signaling pathways.

Adverse health effects in individuals of all ages have been linked to exposure to ambient air contaminants (American Academy of Pediatrics 2004; Jerrett et al. 2005; Pope et al. 2004a). Cardiovascular and respiratory morbidity and mortality are the most common health effects associated with exposure to ambient air contaminants, but other effects that have been linked epidemiologically include adverse fetal development (Dejmek et al. 2000; Liu et al. 2003; Perera et al. 2003) and cancer (Boffetta 2004; Cordier et al. 2004; Vineis et al. 2004). The actual mechanism by which these effects may be produced is frequently unclear, although a substantial body of work has elucidated the relationship between airborne particulate matter (PM) and vasoconstriction (Barclay et al. 2005; Brook et al. 2004; Pope et al. 2004b).

The possibility that ambient air contaminants may be involved in endocrine disruption is an intriguing question (Rogan and Ragan 2003). Several studies have demonstrated the ability of organic contaminants found in ambient air to activate intracellular receptors that are the entry points to signaling pathways implicated in endocrine disruption (Clemons et al. 1998; Vinggaard et al. 2000; Vondracek et al. 2004). In addition to epidemiologic studies that have demonstrated an association between exposure to ambient air contaminants and endocrine-disruptive effects (Jedrychowski et al. 2004; Perera et al. 2003, 2004), *in vivo* studies have shown reproductive effects in rodents exposed via inhalation to diesel exhaust (Furuta et al. 2004; Takeda et al. 2004; Watanabe and Kurita 2001).

Most toxicologic research examining ambient organic air contaminants has focused on contaminants sorbed to PM, yet the specific role that these organic contaminants may play in the biologic effects of ambient air contaminants remains unknown. A large fraction of many airborne chemical classes is present in part or almost exclusively in the gas phase, which has received little if any characterization for biologic activity.

In addition to the substantial challenge of determining the mechanisms underlying health effects from exposure to ambient air, it is important to recognize that not all of the potentially biologically active constituents of organic air contaminants have been isolated and identified. Urban air contains a complex mixture of constituents, of which only a small fraction has been identified (Rogge et al. 1993). Transformation products are rarely analyzed or considered in mass-balance analyses of the activity of organic extracts of ambient air contaminants in receptor-activation assays, even though they exhibit biologic activity (Fertuck et al. 2001; Machala et al. 2004; Wang et al. 2003). Further, exposure to these contaminants is variable and depends on such factors as the proximity to point or nonpoint sources, spatial and temporal variation, and atmospheric conditions.

To address the influence of spatial and temporal factors and to examine the potential biologic activity of the gas phase of organic contaminants in ambient air, we subjected extracts of particulate- and gas-phase ambient air samples, collected from both urban and rural locations in two seasons (winter and summer) over 2 years, to estrogen receptor (ER) chemically activated luciferase expression (CALUX) and aryl hydrocarbon receptor (AHR) CALUX cell bioassays. The CALUX assay is a rapid, sensitive, and well-validated method of assessing the capacity of either mixtures or single compounds to bind and activate the ER and AHR signaling pathways (Denison et al. 2004; Houtman et al. 2004; Rogers and Denison 2000). Previous studies have demonstrated that organic extracts of the particulate phase of ambient air contaminants are capable of activating these pathways in different *in vitro* assays, including CALUX (Hamers et al. 2000), but to our knowledge no study has yet examined the activity of gas-phase extracts.

## Materials and Methods

### Collection and preparation of air samples.

Urban air samples were collected in downtown Toronto, Ontario, Canada, and rural air samples in Egbert, Ontario, Canada, 75-km northeast of Toronto. The downtown site was on the roof of a three-story building located on a heavily traveled street. The rural site was located at ground level at Environment Canada’s Centre for Atmospheric Research. Samples were collected simultaneously at both sites for each sampling period. There were four sampling periods: 20–24 March 2000, 6–16 July 2000, 3–13 March 2001, and 31 July to 8 August 2001. Temperatures during the sampling periods were approximately 10°C for the winter (March) and 25°C for the summer (July–August). High-volume air samplers fitted with polyurethane foam plugs and Teflon filters were used to collect particulate-and gas-phase samples of ambient air. Samplers were run at a flow rate of approximately 1,000 m^3^ per 24 hr. Samples were composited to represent the equivalent of approximately 8,000 m^3^ of air taken over 5–10 days (in shorter sampling periods, two air samplers were deployed simultaneously).

Details of the extraction and cleanup of the air samples were reported previously by Dann (1998). Extracts were split, with 50% going to bioassays and 25% going to chemical analysis of polychlorinated dibenzo-*p*-dioxins/dibenzofurans (PCDDs/PCDFs) and nitro-polycyclic aromatic hydrocarbons (N-PAHs), and 25% going to chemical analysis of PAHs, polychlorinated biphenyls (PCBs), and organochlorine (OC) pesticides. PAHs were quantified by capillary-column gas chromatography/low-resolution mass spectrometry (GC/MS), whereas PCBs and OC pesticides were quantified by gas chromatography with electron capture detection (Harner et al. 2004). PCDDs/PCDFs and N-PAHs were quantified by high-resolution GC and high-resolution MS (Dann 1998). Concentrations were corrected for recoveries only for the PCDDs/PCDFs. Concentrations were not corrected for blanks because analyte concentrations in both method and field blanks were typically < 5% of those in the test samples.

### Reporter cell lines.

The AHR reporter cell line H1L6.1c1, a murine hepatoma cell line (Han et al. 2004), and the ER reporter cell line BG1Luc4E2, a human ovarian carcinoma cell line (Rogers and Denison 2000), are stably transfected cell lines in which luciferase expression is regulated by AHR (H1L6.1c1) or ER (BG1Luc4E2). Cell lines were maintained as described previously by Hodge et al. (2003) and Rogers and Denison (2000).

#### Treatment of H1L6.1c1 cells.

Cells were plated at a density of 6 × 10^4^ cells/well in 12-well plates. After 24 hr, cells were treated with growth medium [α-minimal essential medium (αMEM) plus 10% fetal bovine serum (FBS); media control], growth medium plus 0.1% dimethyl sulfoxide (DMSO; solvent control), reference agonist [10^−7^ M β-naphthoflavone (β-NF)], or various dilutions of the air sample extracts. After 4 hr, the cells were lysed, and luciferase activity assessed as previously described (Garrison et al. 1996) using a commercially available luciferase assay (Promega, Madison, WI, USA). A 4-hr exposure was chosen to minimize any effect of cellular metabolism on the chemical composition of the extract (Windal et al. 2005). Luciferase activity was expressed as relative light units per milligram of cell lysate protein.

#### Treatment of BG1Luc4E2 cells.

Cells were plated at a density of 6 × 10^4^ cells/well in 12-well plates. After 24 hr, the medium was changed to estrogen-reduced medium to minimize the basal expression of luciferase. Estrogen-reduced medium consisted of αMEM without phenol red (Gibco BRL, Burlington, ON, Canada) and 10% charcoal-stripped FBS (Hyclone Inc., Logan, UT, USA). After 48 hr, cells were treated with 17β-estradiol (E_2_)-reduced medium alone (media control), E_2_-reduced media plus 0.1% DMSO (solvent control), reference agonist (10^−10^ M E_2_), or various dilutions of air sample extracts. At 72 hr, the cells were harvested and luciferase activity assessed as described (Rogers and Denison 2000).

### Statistical analyses.

In all cases, protein concentration was determined by the method of Bradford (1976). Curve fitting was performed using GraphPad Prism software (version 3.0; GraphPad Software, San Diego, CA, USA). Principal components analysis (PCA) was performed using SPSS version 11.0 (SPSS, Chicago, IL, USA).

## Results and Discussion

### Chemical analysis.

**Figure 1:**
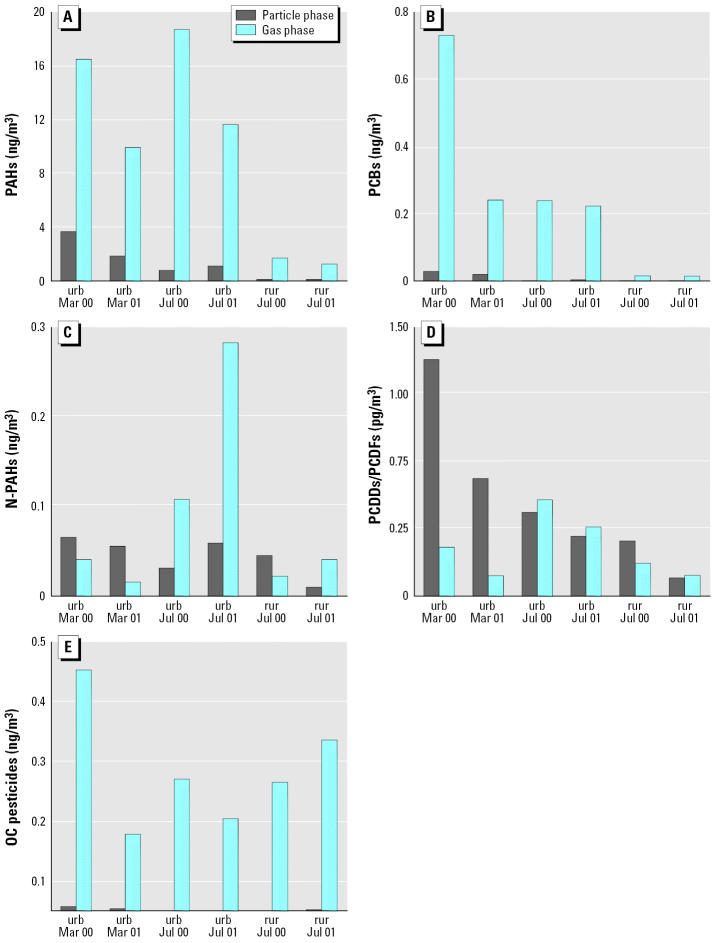
Chemical composition of the ambient air extracts from air samples collected March 2000–July 2001. Abbreviations: rur, rural sample; urb, urban sample. (*A*) PAHs. (*B*) PCBs. (*C*) N-PAHs. (*D*) PCDDs/PCDFs. (*E*) OC pesticides. See “Materials and Methods” for details of experiments.

The concentrations of the sum (∑) of PCDDs/PCDFs (∑PCDDs/PCDFs; picograms per cubic meter of air), ∑PAHs (nanograms per cubic meter), ∑PCBs (nanograms per cubic meter), ∑N-PAHs (nanograms per cubic meter) and ∑OC pesticides (nanograms per cubic meter) in each of the 12 air samples are shown in Figure 1. The PAHs were the most abundant of all the chemical classes we analyzed and were present at concentrations 10^3^ to 10^4^ times greater than ∑PCDDs/PCDFs, and 10–100 times greater than ∑PCBs, ∑N-PAHs, and ∑OC pesticides. These concentrations are similar to those previously reported for other urban centers of North America and Western Europe (Currado and Harrad 2000; Dimashki et al. 2001; Lohmann et al. 2000).

As expected, concentrations of ∑PAHs, ∑PCDDs/PCDFs, ∑N-PAHs, and ∑PCBs in particulate- and gas-phase samples were higher in urban than in rural locations for July samples because of the geographic concentration of emission sources in the urban location. The absence of an urban–rural pattern in total OC pesticide concentrations may be explained by long-range transport of “legacy” pesticides long banned in North America [e.g., DDT (dichlorodiphenyltrichloroethane)] as well as agricultural activities and atmospheric transport of current-use pesticides such as endosulfan and lindane (Gingrich et al. 2001; Harner et al. 2004).

Concentrations of ∑PAHs and ∑PCDDs/PCDFs were similar between March and July samples, indicating temporally consistent sources such as mobile and stationary combustion (Lee et al. 2003). ∑OC pesticide concentrations were also similar with season, consistent with regional and long-range transport (Harner et al. 2004). Contrary to expectation, ∑PCBs did not show seasonal differences (Halsall et al. 1995), suggesting that regional atmospheric transport may be the source. ∑N-PAH concentrations were greatest in July, which suggests increased formation of these chemicals resulting from photochemical processes (Atkinson and Arey 1994).

The compounds we analyzed have a wide range of volatilities and varied in their distribution between gas and particulate phases. With the exceptions of ∑PCDDs/PCDFs and March ∑N-PAHs, most compounds were found in gas-phase samples. Not including the PCBs, the five most abundant compounds and congeners in the particulate-and gas-phase samples accounted for 60–95% of the total amount of compound in each class (Table 1). Also, there was no overlap between the most abundant PCBs in the particulate phase and the most abundant PCBs in the gas phases, whereas the most abundant compounds for each of the other chemical classes were similar in both gas and particulate phases. However, the overall concentration of PAHs and OC pesticides were 10 times greater in the gas phase than in the particulate phase, and the reverse was true for PCDDs/PCDFs.

**Figure 2:**
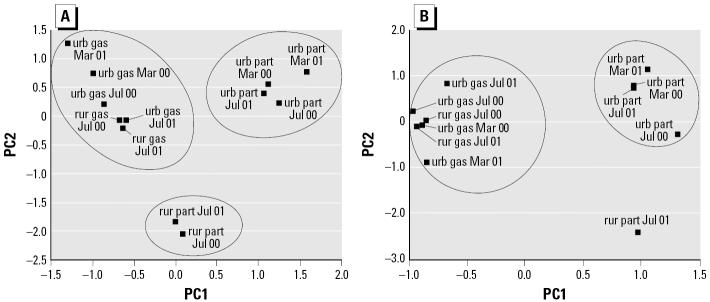
PCA of PAH and PCB chemical data. Abbreviations: part, particulate; rur, rural sample; urb, urban sample. (*A*) PAHs; the first two components accounted for 87% of the variance. (*B*) PCBs; the first two components accounted for 60% of the variance.

We performed PCA to identify major contributions to variance in the chemical composition of the samples. Data were autoscaled to unit variance, and the analysis included only those compounds/congeners present in both gas and particulate phases. As shown in Figure 2A, the first two components accounted for 87% of the variance for PAHs. Three clusters are evident in the plot for PAHs: all gas-phase samples in the upper left quadrant, which separate from urban particulate phase samples along PC1, and rural particulate phase samples along PC2. Within the cluster of gas-phase samples, July versus March samples separated along PC2. The similarity of urban and rural gas-phase samples relative to their respective particulate-phase samples is likely because of the greater transport distance of gas-phase versus particulate-phase PAHs.

The concentrations of the gas-phase PAHs anthracene, acenaphthene, fluorene, and phenanthrene distinguished the gas phase from urban and rural particulate phases. Variance in rural particulate-phase samples was explained by concentrations of chrysene, pyrene, and fluoranthene, whereas the variance in urban particulate-phase samples was explained by triphenylene, benzo[*a*]fluorene, and benzo[*b*]fluorene.

Samples in the PCA analysis of the PCB data clustered in the same pattern as for PAHs: all gas phase, urban particulate phase, and rural particulate phase (Figure 2B). The first two components accounted for 60% of the variance among samples. The rural particulate-phase July 2000 sample was excluded because of the high-number congeners that were below detection. Variance of the gas-phase samples was explained by trichlorinated and tetrachlorinated congeners (e.g., PCB congeners 16/32, 49, 70/76), whereas variance in urban particulate-phase samples was explained by hexachlorinated and heptachlorinated congeners (e.g., PCB congeners 138, 183, 170).

### Biologic activity of air samples in cells in culture.

Both gas- and particulate-phase extracts induced significant activation of ER-and AHR-dependent luciferase gene expression. The distinctions in chemical composition between phase, site, and season were not reflected in the results of the CALUX assays, suggesting the aggregate biologic activity of the different congeners in each extract is similar.

As described above, PAHs were the most abundant of the chemical classes analyzed, present at concentrations approximately three to four orders of magnitude greater than those of the potent AHR agonists PCDDs/PCDFs and one to two orders of magnitude greater than concentrations of the PCBs. These relative amounts are an important factor in the ultimate potency of these extracts. Less than additive, weak agonist interactions are observed with mixtures in *in vitro* AHR assays, where the concentration of potent agonists is significantly lower than those of more moderate agonists (Safe 1997–1998). Thus, the activity of each extract is influenced by these interactions and reflects the composition of the PAH fraction rather than the PCDD/F and PCB fractions.

### AHR reporter cell line.

**Figure 3:**
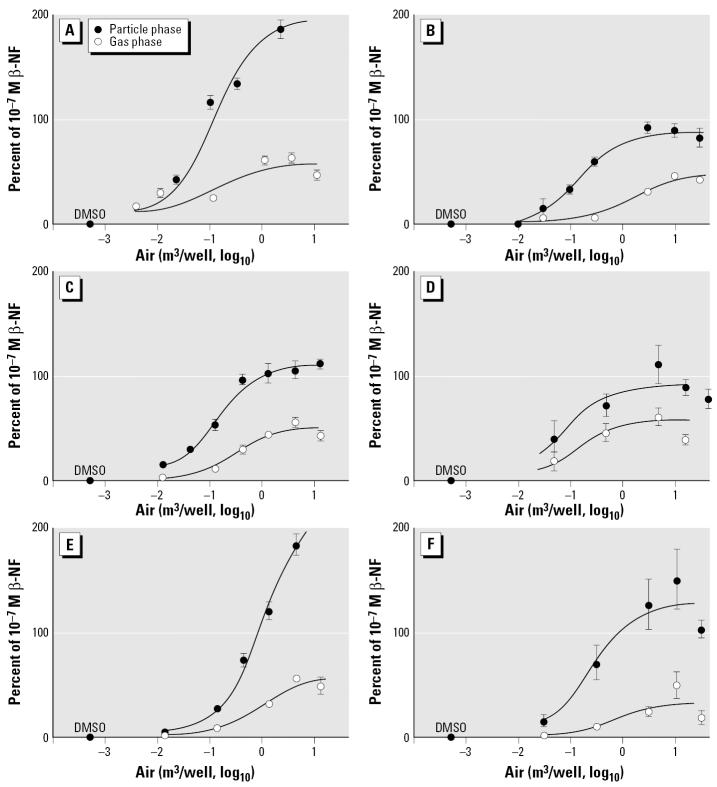
Dose–response curves for AHR-responsive cells treated with varying concentrations of air extracts (m^3^ air/well) of gas-phase and particulate-phase ambient air samples. (*A*) Urban sample, March 2000. (*B*) Urban sample, March 2001. (*C*) Urban sample, July 2000. (*D*) Urban sample, July 2001. (*E*) Rural sample, July 2000. (*F*) Rural sample, July 2001. Activity is expressed as percentage of the response relative to 10^−7^ M β-NF. Data represent mean ± SD from three separate experiments. Dose–response curves were generated using GraphPad Prism software.

Figure 3 shows the results of extracts in the H1L6.1c1 cell line, grouped by date and site. Results are normalized to protein content in cell extracts and expressed as a percentage of the response in the cell line to 10^−7^ M β-NF, which represented the 95th percentile of the β-NF dose–response curve and 100% of the response to 10^−9^ M 2,3,7,8-tetrachlorodibenzo-*p*-dioxin (data not shown).

All extracts induced luciferase expression in a concentration-dependent manner. In most cases, upper plateaus of response were observed, allowing for comparison of efficacy between extracts. In all cases, gas-phase extracts were less efficacious than the corresponding particulate-phase extracts for the same sampling dates and locations. If the activity of the extracts was driven by the concentration of PAHs, the higher activity of the particulate-phase extracts could be explained by the relatively higher affinity for the AHR of PAHs in this fraction, compared with those in the gas phase (Table 1) (Piskorska-Pliszczynska et al. 1986; Till et al. 1999). Given the relatively large amounts of PAHs in the extracts, we expect this class of contaminants to be at least partly responsible for the AHR-dependent inducing activity observed. Moreover, because of the short duration (4 hr) of the cells’ exposure to the mixture, the PAH concentrations would not have been diminished by metabolism.

Ranking the relative potency of the extracts was challenging. The concentration–response curves were not parallel, and the maximum responses of some extracts exceeded the response to a maximally inducing concentration of 10^−7^ M β-NF. The reasons for these results are not known; however, others have reported this phenomenon. Inhibition of AHR degradation results in superinduction of AHR-inducible genes (Ma 2002). Alternatively, activation of other signaling pathways may augment AHR function (Seidel et al. 2001) or result in a synergistic increase in AHR-dependent gene expression (Chen and Tukey 1996; Long et al. 1998). The complex mixture that constitutes the gas-and particulate-phase air extracts could be activating any or all of these mechanisms.

The lower efficacy of gas-phase extracts compared with particulate-phase extracts was the only statistically significant observation (unpaired *t*-test, *p* = 0.005) between origin of sample and biologic activity. Similar analyses between seasons or locations and efficacy were not statistically significant despite the separation of rural and particulate-phase samples in the PCA. The lack of association between location and efficacy has been reported by others (Binkovà et al. 1999).

It was not possible to calculate a true EC_50_ (concentration which produced 50% of the maximum) value for all samples; therefore, to aid comparison of one sample with another, Table 2 shows the concentration of air that elicited 20% and 50% of the response to 10^−7^ M β-NF in the AHR reporter cell line. Using these values as a measure of relative potency, Table 2 demonstrates that maximum response to a sample can differ for samples with similar relative potency. For example, the urban particulate extract of March 2000 and the urban particulate extract of July 2000 have similar activity at 20% or 50% of the response to 10^−7^ M β-NF, yet the maximum activity of each sample is quite different. Similarly, the rural particulate extract of July 2000 has similar relative potency to the urban gas-phase extract of July 2001, yet the maximum responses are quite dissimilar. In contrast, the rural gas-phase extract of July 2001 and the urban gas-phase extract of March 2001 are similar in all respects.

These divergent patterns of concentration response are not unexpected. Ligand potency depends not only on the affinity of the receptor for the ligand but also on the ability of the ligand–receptor complex to bind additional transcription factors and elicit a response (Hestermann et al. 2000; Kohn and Melnick 2002). The dose–response curve for each extract is the outcome of the complex interplay of these events. The net effect, that is, the dose–response curve, will depend on the relative agonist–antagonist properties, which are different for each sample in this study (Table 1).

### ER reporter cell line.

**Figure 4:**
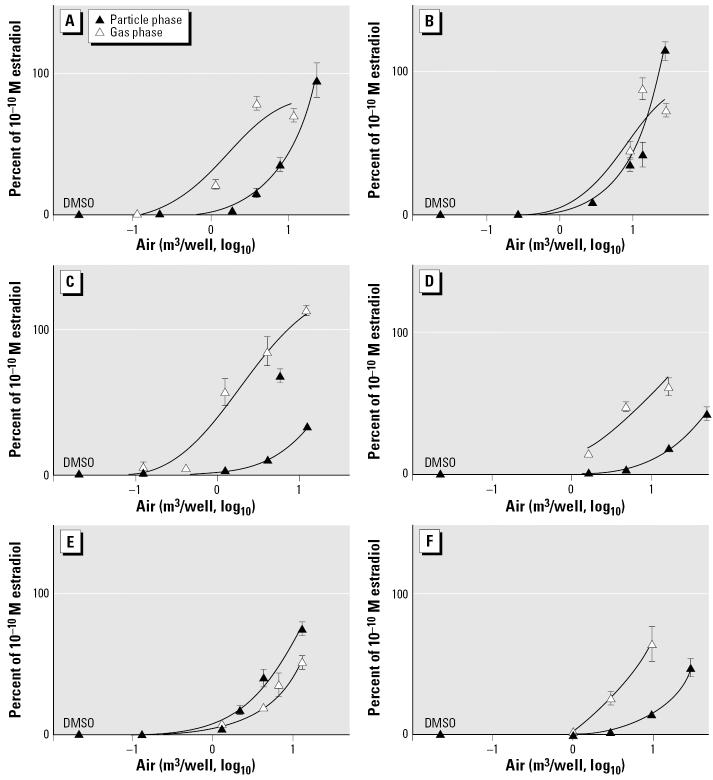
Dose–response curves for ER-responsive cells treated with varying concentrations of air extracts (m^3^ air/well) of gas-phase and particulate-phase ambient air samples. (*A*) Urban sample, March 2000. (*B*) Urban sample, March 2001. (*C*) Urban sample, July 2000. (*D*) Urban sample, July 2001. (*E*) Rural sample, July 2000. (*F*) Rural sample, July 2001. Activity is expressed as percentage of the response to 10^−10^ M estradiol. Data represent mean ± SD from three separate experiments. Dose–response curves were generated using GraphPad Prism software.

Results of experiments using the extracts in the ER reporter cell line BG1Luc4E2 are shown in Figure 4, where concentration–response data are grouped by site and date. Results were normalized to protein content in cell extracts and expressed as a percentage of the maximum response of 10^−10^ M E_2_.

In contrast to the results obtained in the AHR reporter cell line, the activity of gas-phase extracts, judged by magnitude of induction, frequently exceeded that of particulate-phase extracts in the ER reporter cell line (see, e.g., the gas- vs. particulate-phase extracts for urban March and July 2000, Figure 4A,C). This result is of interest because, as described above, most research examining the activity of organic contaminants of ambient air has focused on particulate-phase extracts. Our results indicate that the gas phase may have activity equal or greater than that of the particulate phase in an ER reporter assay. Table 3 reports the concentration of air at which 50% and 20% of the activity of the reference standard was induced in the ER reporter cell line. As observed for AHR responses, there was no apparent difference in activity between rural and urban sites or between seasons.

Possible sources of estrogenic activity in the ambient air extracts include PAH, PCBs, N-PAHs, and OC pesticides. Various PAHs and PCBs and their metabolites (Fujimoto et al. 2003; Furuta et al. 2004; Pliskova et al. 2005) as well as OC pesticides (Kojima et al. 2004) are reported to be estrogenic. Complicating interpretation of the estrogenic activity, however, is the fact that some PAHs and PCBs and their metabolites also exhibit antiestrogenic activity (Arcaro et al. 1999; Connor et al. 1997; Kramer et al. 1997).

Cellular metabolism in the BG1Luc4E2 cell line includes the cytochrome P450 enzymes, which yield hydroxyl metabolites of PCBs, PAHs, and N-PAHs. Hydroxylated PCBs have been shown to exhibit estrogenic activity in ER-CALUX assays and, in some cases, are more potent than the parent PCBs (Layton et al. 2002; Nesaretnam et al. 1996). Hydroxy-PAHs have also been shown to bind and/or activate ER (Charles et al. 2000; Fertuck et al. 2001). Additional classes of estrogenic chemicals not analyzed in our studies but likely to be present in the extracts include phthalate esters, which have been shown to be weak ER agonists (Zacharewski et al. 1998).

## Conclusions

To our knowledge, this is the first study to examine the dioxin-like and estrogenic activity of organic extracts of the gas phase of ambient air. Our results not only suggest that mechanistic studies of the health effects of ambient air need to consider both phases as having the potential to induce and/or inhibit AHR and ER signaling pathways but also suggest that the relationship between chemical composition and biologic activity is highly complex. Two of the expected factors driving variation in chemical composition between our samples (phase and location) were mathematically detectable in the PCA. However, the results for urban versus rural location were not discernible in the results of the highly sensitive CALUX bioassays. This may be due to unidentified compounds, nonlinearity between the magnitude of differences in chemical concentration and the response in the CALUX assay, interactions between chemicals and cellular factors, the presence of both agonists and antagonists in the sample, or, more likely, some combination of the above factors.

There is another possibility, however. The fact that an *in vivo* study comparing the biologic activity (AHR mediated) of organic extracts of PM_10_ along an urban–rural gradient and between seasons also did not find significant locational differences, despite differences in ambient air concentrations of AHR or ER agonists (Binkovà et al. 2003), may be because the concentration response of exposure to mixtures of AHR agonists is not sigmoidal due to the impact of weak agonist interactions (Kohn and Melnick 2002). That is to say, the biologic effects of exposure to mixtures of relatively lower concentrations may not be exceeded by those of exposure to mixtures at relatively higher concentrations.

Interestingly, seasonal and locational differences have been observed in some epidemiologic studies comparing different biomarkers and end points with ambient air composition (Dejmek et al. 2000; Perera et al. 2003). The absence of any similar finding in this *in vitro* study may be due to the high sensitivity of the CALUX assay and its “remoteness” as a receptor assay looking at a specific aspect of one signaling pathway in contrast to the more physiologically complicated *in vivo* effects examined in epidemiology or other toxicologic studies.

One unanswered question is the relationship between the biologic effects observed in these cell-based assays and inhalation exposures in humans. The average daily intake of air ranges from about 2 m^3^/day in very young infants to about 24 m^3^/day in adults (Health Canada 1995). Based on these intake rates, an individual could be exposed to levels at which we observe an effect *in vitro* and are sufficient to elicit a response in some biologic systems (Bateson and Schwartz 2004; Pope et al. 2004b; Vineis et al. 2004). However, an important consideration is that uptake characteristics are very different between a cell in culture (likely 100%) and inhalation (much less than 100%). Whether or not there is a sustained effect in humans remains to be determined.

## Figures and Tables

**Table 1:** Mean percentage of sample composed of the five most abundant compounds listed in descending order.

Compounds	Gas phase	Particulate phase
PAHs	95% of total: phenanthrene, fluoranthene, pyrene, fluorene, anthracene, 2-methyl fluorine	60% of total: benzo[*g*,*h*,*i*]perylene, benzo[*b*]fluoranthene, fluoranthene, pyrene, indenol[1,2,3-*c*,*d*]pyrene
PCBs	13% of total: PCB congeners 52, 95, 31, 18, and 101	8% of total: PCB congeners 138, 153, 8/5, 180, 149
N-PAHs	99% of total: 9-nitroanthracene, 2-nitrofluoranthene, 1-nitropyrene, 9-nitrophenanthrene, 7-nitrobenz[*a*]anthracene	100% of total: 9-nitroanthracene, 2-nitrofluoranthene, 1-nitropyrene, 7-nitrobenz[*a*]anthracene, 6-nitrobenzo[*a*]pyrene
PCDDs/PCDFs	88% of total: 2,3,7,8,-tetraCDF, octaCDD, 1,2,3,4,6,7,8,-hepta-CDD, 1,2,3,4,6,7,8,-heptaCDF, 2,3,4,7,8,-pentaCDF, 1,2,3,4,7,8-hexaCDF	94% of total: OCDD, 1,2,3,4,6,7,8-heptaCDD, 1,2,3,4,6,7,8-heptaCDF, octaCDF
OC pesticides	82% of total: endosulfan, γ -HCH, *p*,*p*′-DDE, dieldrin, α -HCH	82% of total: endosulfan, *p*,*p*′-DDT, *p*,*p*′-DDE, dieldrin, γ -HCH

Abbreviations: CDD, chlorinated dibenzodioxin; CDF, chlorinated dibenzofuran; DDE, dichlorodiphenyldichloroethylene; HCH, hexachlorcyclohexane.

**Table 2:** Apparent potency of samples in AHR reporter gene assay at different induction levels and maximum induction, in descending order.

Sample	20% of 10^−7^ M β-NF (m^3^)	Sample	50% of 10^−7^ M β-NF (m^3^)	Sample	Efficacy (% of β-NF)
urb part Mar 00	0.01	urb part Mar 00	0.04	urb part Mar 00	No plateau
urb part Jul 01	0.02	urb part Jul 01	0.08	rur part Jul 00	No plateau
urb part Jul 00	0.02	urb part Jul 00	0.09	rur part Jul 01	129
urb gas Mar 00	0.03	rur part Jul 01	0.14	urb part Jul 00	110
rur part Jul 01	0.04	urb part Mar 01	0.18	urb part Jul 01	92
urb part Mar 01	0.04	rur Jul part 00	0.28	urb part Mar 01	87
urb gas Jul 01	0.06	urb gas Jul 01	0.58	urb gas Jul 01	60
rur part Jul 00	0.09	urb gas Mar 00	0.66	urb gas Mar 00	58
urb gas Jul 00	0.19	rur gas Jul 00	5.00	rur gas Jul 00	58
rur gas Jul 00	0.41	urb gas Jul 00	6.32	urb gas Jul 00	52
rur gas Jul 01	0.9	rur gas Jul 01	—a	urb gas Mar 01	48
urb gas Mar 01	1.02	urb gas Mar 01	—a	rur gas Jul 01	33

Abbreviations: part, particulate; rur, rural sample; urb, urban sample.

a Sample did not elicit 50% of the activity of 10^−7^ M β-NF.

**Table 3:** Apparent potency of samples in ER reporter gene assay at different induction levels, in descending order.

Sample	20% of 10^−10^ estradiol (m^3^)	Sample	50% of 10^−10^ estradiol (m^3^)
urb gas Jul 00	0.39	urb gas Jul 00	1.26
urb gas Mar 00	0.62	urb gas Mar 00	2.25
urb gas Jul 01	1.90	rur gas Jul 01	6.23
rur gas Jul 01	2.27	rur part Jul 00	6.92
rur part Jul 00	2.32	urb gas Jul 01	7.65
urb gas Mar 01	2.90	urb gas Mar 01	9.48
rur gas Jul 00	3.97	urb part Mar 00	11.23
urb part Mar 00	4.58	rur gas Jul 00	12.45
urb part Mar 01	5.28	urb part Mar 01	12.50
urb part Jul 00	7.26	rur part Jul 01	—a
rur part Jul 01	12.25	urb part Jul 00	—a
urb part Jul 01	18.47	urb part Jul 01	—a

Abbreviations: part, particulate; rur, rural sample; urb, urban sample.

a Sample did not elicit 50% of the activity of 10^−10^ estradiol.

## References

[b1-ehp0114-000697] American Academy of Pediatrics Committee on Environmental Health (2004). Ambient air pollution: health hazards to children. Pediatrics.

[b2-ehp0114-000697] Arcaro KF, O’Keefe PW, Yang Y, Clayton W, Gierthy JF (1999). Antiestrogenicity of environmental polycyclic aromatic hydrocarbons in human breast cancer cells. Toxicology.

[b3-ehp0114-000697] Atkinson R, Arey J (1994). Atmospheric chemistry of gas-phase polycyclic aromatic-hydrocarbons: formation of atmospheric mutagens. Environ Health Perspect.

[b4-ehp0114-000697] Barclay J, Hillis G, Ayres J (2005). Air pollution and the heart: cardiovascular effects and mechanisms. Toxicol Rev.

[b5-ehp0114-000697] Bateson TF, Schwartz J (2004). Who is sensitive to the effects of particulate air pollution on mortality? A case-crossover analysis of effect modifiers. Epidemiology.

[b6-ehp0114-000697] Binkovà B, Cerná M, Pastorková A, Jelínek R, Benes I, Novák J (2003). Biological activities of organic compounds adsorbed onto ambient air particles: comparison between the cities of Teplice and Prague during the summer and winter seasons 2000–2001. Mutat Res.

[b7-ehp0114-000697] Binkovà B, Vesely D, Vesela D, Jelinek R, Sram RJ (1999). Genotoxicity and embryotoxicity of urban air particulate matter collected during winter and summer period in two different districts of the Czech Republic. Mutat Res.

[b8-ehp0114-000697] Boffetta P (2004). Epidemiology of environmental and occupational cancer. Oncogene.

[b9-ehp0114-000697] Bradford MM (1976). A rapid and sensitive method for the quantitation of microgram quantities of protein utilizing the principle of protein-dye binding. Anal Biochem.

[b10-ehp0114-000697] Brook RD, Franklin B, Cascio W, Hong Y, Howard G, Lipsett M (2004). Air pollution and cardiovascular disease: a statement for healthcare professionals from the Expert Panel on Population and Prevention Science of the American Heart Association. Circulation.

[b11-ehp0114-000697] Charles GD, Bartels MJ, Zacharewski TR, Gollapudi BB, Freshour NL, Carney EW (2000). Activity of benzo[*a*]pyrene and its hydroxylated metabolites in an estrogen-receptor alpha reporter gene assay. Toxicol Sci.

[b12-ehp0114-000697] Chen YH, Tukey RH (1996). Protein kinase C modulates regulation of the CYP1A1 gene by the aryl hydrocarbon receptor. J Biol Chem.

[b13-ehp0114-000697] Clemons JH, Allan LM, Marvin CH, Wu Z, McCarry BE, Bryant DW (1998). Evidence of estrogen- and TCDD-like activities in crude and fractionated extracts of PM_10_ air particulate material using *in vitro* gene expression assays. Environ Sci Technol.

[b14-ehp0114-000697] Connor K, Ramamoorthy K, Moore M, Mustain M, Chen I, Safe S (1997). Hydroxylated polychlorinated biphenyls (PCBs) as estrogens and antiestrogens: structure-activity relationships. Toxicol Appl Pharmacol.

[b15-ehp0114-000697] Cordier S, Monfort C, Filippini G, Preston-Martin S, Lubin F, Mueller BA (2004). Parental exposure to polycyclic aromatic hydrocarbons and the risk of childhood brain tumors: the SEARCH International Childhood Brain Tumor Study. Am J Epidemiol.

[b16-ehp0114-000697] Currado GM, Harrad S (2000). Factors influencing atmospheric concentrations of polychlorinated biphenyls in Birmingham, U.K. Environ Sci Technol.

[b17-ehp0114-000697] Dann T 1998. Ambient air measurements of polycyclic aromatic hydrocarbons (PAH), polychlorinated dibenzo-*p*-dioxins (PCDD) and polychlorinated dibenzofurans in Canada (1987–1997). AAQD 97–93. Ottawa, Ontario, Canada:Analysis and Air Quality Division, Environment Canada.

[b18-ehp0114-000697] Dejmek J, Solansky I, Benes I, Lenicek J, Srám RJ (2000). The impact of polycylic aromatic hydrocarbons and fine particles on pregnancy outcome. Environ Health Perspect.

[b19-ehp0114-000697] Denison MS, Zhao B, Baston DS, Clark GC, Murata H, Han D-H (2004). Recombinant cell bioassay systems for the detection and relative quantitation of halogenated dioxins and related chemicals. Talanta.

[b20-ehp0114-000697] Dimashki M, Lim LH, Harrison RM, Harrad S (2001). Temporal trends, temperature dependence, and relative reactivity of atmospheric polycyclic aromatic hydrocarbons. Environ Sci Technol.

[b21-ehp0114-000697] Fertuck KC, Kumar S, Sikka HC, Matthews JB, Zacharewski TR (2001). Interaction of PAH-related compounds with the alpha and beta isoforms of the estrogen receptor. Toxicol Lett.

[b22-ehp0114-000697] Fujimoto T, Kitamura S, Sanoh S, Sugihara K, Yoshihara S, Fujimoto N (2003). Estrogenic activity of an environmental pollutant, 2-nitrofluorene, after metabolic activation by rat liver microsomes. Biochem Biophys Res Commun.

[b23-ehp0114-000697] Furuta C, Suzuki AK, Taneda S, Kamata K, Hayashi H, Mori Y (2004). Estrogenic activities of nitrophenols in diesel exhaust particles. Biol Reprod.

[b24-ehp0114-000697] Garrison PM, Tullis K, Aarts JM, Brouwer A, Giesy JP, Denison MS (1996). Species-specific recombinant cell lines as bioassay systems for the detection of 2,3,7,8-tetrachlorodibenzo-*p*-dioxin-like chemicals. Fundam Appl Toxicol.

[b25-ehp0114-000697] Gingrich SE, Diamond ML, Stern GA, McCarry BE (2001). Atmospherically derived organic surface films along an urban-rural gradient. Environ Sci Technol.

[b26-ehp0114-000697] Halsall CJ, Lee RGM, Coleman PJ, Burnett V, Harding-Jones P, Jones KC (1995). PCBs in U.K. air. Environ Sci Technol.

[b27-ehp0114-000697] Hamers T, van Schaardenburg MD, Felzel EC, Murk AJ, Koeman JH (2000). The application of reporter gene assays for the determination of the toxic potency of diffuse air pollution. Sci Total Environ.

[b28-ehp0114-000697] Han D, Nagy SR, Denison MS (2004). Comparison of recombinant cell bioassays for the detection of Ah receptor agonists. Biofactors.

[b29-ehp0114-000697] Harner T, Shoeib M, Diamond M, Stern G, Rosenberg B (2004). Using passive air samplers to assess urban-rural trends for persistent organic pollutants. 1. Polychlorinated biphenyls and organochlorine pesticides. Environ Sci Technol.

[b30-ehp0114-000697] Health Canada 1995. Investigating Human Exposure to Contaminants in the Environment: A Handbook for Exposure Calculations. Ottawa, Ontario, Canada:Health Canada.

[b31-ehp0114-000697] Hestermann EV, Stegeman JJ, Hahn ME (2000). Relative contributions of affinity and intrinsic efficacy to aryl hydrocarbon receptor ligand potency. Toxicol Appl Pharmacol.

[b32-ehp0114-000697] Hodge EM, Diamond ML, McCarry BE, Stern GA, Harper PA (2003). Sticky windows: chemical and biological characteristics of the organic film derived from particulate and gas-phase air contaminants found on an urban impervious surface. Arch Environ Contam Toxicol.

[b33-ehp0114-000697] Houtman CJ, Cenijn PH, Hamers T, Lamoree MH, Legler J, Murk AJ (2004). Toxicological profiling of sediments using in vitro bioassays, with emphasis on endocrine disruption. Environ Toxicol Chem.

[b34-ehp0114-000697] Jedrychowski W, Bendkowska I, Flak E, Penar A, Jacek R, Kaim I (2004). Estimated risk for altered fetal growth resulting from exposure to fine particles during pregnancy: an epidemiologic prospective cohort study in Poland. Environ Health Perspect.

[b35-ehp0114-000697] Jerrett M, Burnett RT, Ma R, Pope CA, Krewski D, Newbold KB (2005). Spatial analysis of air pollution and mortality in Los Angeles. Epidemiology.

[b36-ehp0114-000697] Kohn MC, Melnick RL (2002). Biochemical origins of the non-monotonic receptor-mediated dose-response. J Mol Endocrinol.

[b37-ehp0114-000697] Kojima H, Katsura E, Takeuchi S, Niiyama K, Kobayashi K (2004). Screening for estrogen and androgen receptor activities in 200 pesticides by *in vitro* reporter gene assays using Chinese hamster ovary cells. Environ Health Perspect.

[b38-ehp0114-000697] Kramer VJ, Helferich WG, Bergman Å, Klasson-Wehler, Giesy JP (1997). Hydroxylated polychlorinated biphenyl metabolites are anti-estrogenic in a stably transfected human breast adenocarcinoma (MCF-7) cell line. Toxicol Appl Pharmacol.

[b39-ehp0114-000697] Layton AC, Sanseverino J, Gregory BW, Easter JP, Sayler GS, Schultz TW (2002). *In vitro* estrogen receptor binding of PCBs: measured activity and detection of hydroxylated metabolites in a recombinant yeast assay. Toxicol Appl Pharmacol.

[b40-ehp0114-000697] Lee PKH, Brook JR, Dabek-Zlotorzynska E, Mabury SA (2003). Identification of the major sources contributing to PM_2.5_ observed in Toronto. Environ Sci Technol.

[b41-ehp0114-000697] Liu S, Krewski D, Shi Y, Chen Y, Burnett RT (2003). Association between gaseous ambient air pollutants and adverse pregnancy outcomes in Vancouver, Canada. Environ Health Perspect.

[b42-ehp0114-000697] Lohmann R, Harner T, Thomas GO, Jones KC (2000). A comparative study of the gas-particle partitioning of PCDD/Fs, PCBs, and PAHs. Environ Sci Technol.

[b43-ehp0114-000697] Long WP, Pray-Grant M, Tsai JC, Perdew GH (1998). Protein kinase C activity is required for aryl hydrocarbon receptor pathway-mediated signal transduction. Mol Pharmacol.

[b44-ehp0114-000697] Ma Q (2002). Induction and superinduction of 2,3,7,8-tetrachlorodibenzo-*p*-dioxin-inducible poly(ADP-ribose) polymerase: role of the aryl hydrocarbon receptor/aryl hydrocarbon receptor nuclear translocator transcription activation domains and a labile transcription repressor. Arch Biochem Biophys.

[b45-ehp0114-000697] Machala M, Blaha L, Lehmler HJ, Pliskova M, Majkova Z, Kapplova P (2004). Toxicity of hydroxylated and quinoid PCB metabolites: inhibition of gap junctional intercellular communication and activation of aryl hydrocarbon and estrogen receptors in hepatic and mammary cells. Chem Res Toxicol.

[b46-ehp0114-000697] Nesaretnam K, Corcoran D, Dils RR, Darbre P (1996). 3,4,3′,4′-Tetrachlorobiphenyl acts as an estrogen *in vitro* and *in vivo*. Mol Endocrinol.

[b47-ehp0114-000697] Perera FP, Rauh V, Tsai WY, Kinney P, Camann D, Barr D (2003). Effects of transplacental exposure to environmental pollutants on birth outcomes in a multiethnic population. Environ Health Perspect.

[b48-ehp0114-000697] Perera FP, Rauh V, Whyatt RM, Tsai WY, Bernert JT, Tu YH (2004). Molecular evidence of an interaction between prenatal environmental exposures and birth outcomes in a multiethnic population. Environ Health Perspect.

[b49-ehp0114-000697] Piskorska-Pliszczynska J, Keys B, Safe S, Newman MS (1986). The cytosolic receptor binding affinities and AHH induction potencies of 29 polynuclear aromatic hydrocarbons. Toxicol Lett.

[b50-ehp0114-000697] Pliskova M, Vondracek J, Canton RF, Nera J, Kocan A, Petrik J (2005). Impact of polychlorinated biphenyls contamination on estrogenic activity in human male serum. Environ Health Perspect.

[b51-ehp0114-000697] Pope CA, Burnett RT, Thurston GD, Thun MJ, Calle EE, Krewski D (2004a). Cardiovascular mortality and long-term exposure to particulate air pollution: epidemiological evidence of general pathophysiological pathways of disease. Circulation.

[b52-ehp0114-000697] Pope CA, Hansen ML, Long RW, Nielsen KR, Eatough NL, Wilson WE (2004b). Ambient particulate air pollution, heart rate variability, and blood markers of inflammation in a panel of elderly subjects. Environ Health Perspect.

[b53-ehp0114-000697] Rogan WJ, Ragan NB (2003). Evidence of effects of environmental chemicals on the endocrine system in children. Pediatrics.

[b54-ehp0114-000697] Rogers JM, Denison MS (2000). Recombinant cell bioassays for endocrine disruptors: development of a stably transfected human ovarian cell line for the detection of estrogenic and anti-estrogenic chemicals. In Vitro Mol Toxicol.

[b55-ehp0114-000697] Rogge WF, Hildemann LM, Mazurek MA, Cass GR, Simoneit BRT (1993). Quantification of urban organic aerosols at a molecular level—identification, abundance and seasonal variation. Atmos Environ A.

[b56-ehp0114-000697] Safe S (1997). –1998. Limitations of the toxic equivalency factor approach for risk assessment of TCDD and related compounds. Teratog Carcinog Mutagen.

[b57-ehp0114-000697] Seidel SD, Winters GM, Rogers WJ, Ziccardi MH, Li V, Keser B (2001). Activation of the Ah receptor signaling pathway by prostaglandins. J Biochem Mol Toxicol.

[b58-ehp0114-000697] Takeda K, Tsukue N, Yoshida S (2004). Endocrine-disrupting activity of chemicals in diesel exhaust and diesel exhaust particles. Environ Sci.

[b59-ehp0114-000697] Till M, Riebniger D, Schmitz H-J, Schrenk D (1999). Potency of various polycyclic aromatic hydrocarbons as inducers of CYP1A1 in rat hepatocyte cultures. Chem Biol Interact.

[b60-ehp0114-000697] Vineis P, Forastiere F, Hoek G, Lipsett M (2004). Outdoor air pollution and lung cancer: recent epidemiologic evidence. Int J Cancer.

[b61-ehp0114-000697] Vinggaard AM, Hnida C, Larsen JC (2000). Environmental polycyclic aromatic hydrocarbons affect androgen receptor activation in vitro. Toxicology.

[b62-ehp0114-000697] Vondracek J, Chramostova K, Pliskova M, Blaha L, Brack W, Kozubik A (2004). Induction of aryl hydrocarbon receptor-mediated and estrogen receptor-mediated activities, and modulation of cell proliferation by dinaphthofurans. Environ Toxicol Chem.

[b63-ehp0114-000697] Wang J, Wu W, Henkelmann B, You L, Kettrup A, Schramm K-W (2003). Presence of estrogenic activity from emission of fossil fuel combustion as detected by a recombinant yeast bioassay. Atmos Environ.

[b64-ehp0114-000697] Watanabe N, Kurita M (2001). The masculinization of the fetus during pregnancy due to inhalation of diesel exhaust. Environ Health Perspect.

[b65-ehp0114-000697] Windal I, Denison MS, Birnbaum LS, Van Wouwe N, Baeyens W, Goeyens L (2005). Chemically activated luciferase gene expression (CALUX) cell bioassay analysis for the estimation of dioxin-like activity: critical parameters of the CALUX procedure that impact assay results. Environ Sci Technol.

[b66-ehp0114-000697] Zacharewski TR, Meek MD, Clemons JH, Wu ZF, Fielden MR, Matthews JB (1998). Examination of the *in vitro* and *in vivo* estrogenic activities of eight commercial phthalate esters. Toxicol Sci.

